# Development of an influenza virus protein microarray to measure the humoral response to influenza virus infection in mallards

**DOI:** 10.1038/emi.2017.98

**Published:** 2017-12-06

**Authors:** Philip Meade, Neus Latorre-Margalef, David E Stallknecht, Florian Krammer

**Affiliations:** 1 Department of Microbiology, Icahn School of Medicine at Mount Sinai, New York, NY 10029, USA; 2 Graduate School of Biomedical Sciences, Icahn School of Medicine at Mount Sinai, New York, NY 10029, USA; 3 Department of Biology, Lund University, Lund SE-221-00, Sweden; 4 Southeastern Cooperative Wildlife Disease Study, College of Veterinary Medicine, University of Georgia, Athens, GA 30602, USA

**Keywords:** avian influenza, ducks, influenza, mallards, serology

## Abstract

Avian influenza viruses pose a serious zoonotic threat, in part because current seasonal influenza virus vaccines only offer strain-specific protection, instead of heterosubtypic or universal protection against influenza virus infection. Understanding the humoral response to vaccination and natural infection in the broadest context possible is important to developing defenses against influenza zoonosis. Protein microarrays are a novel platform well suited to assaying the humoral immune response broadly and efficiently. We developed an influenza virus protein microarray (IVPM) that could be used to assay sera from many species, including humans. Waterfowl such as mallard ducks are natural reservoirs for many influenza A viruses, but their humoral immune response to infection is poorly understood. To establish this technology, we assayed sera from mallards experimentally infected with two low-pathogenic common avian influenza viruses (H3N8 and H4N5) for reactivity to influenza virus hemagglutinin (HA) by IVPM. The IVPM results correlated well with results from an established enzyme-linked immunosorbent assay, supporting the validity of the IVPM as a serological assay in influenza virus research. Interestingly, successive infection with H3N8 followed by H4N5 virus in mallard ducks induced antibodies that were broadly reactive against group 2 hemagglutinins. We also analyzed sera from wild mallards and observed serological evidence for infection in those sera. With serological information, it may be possible to infer infection history of wild avian species and gain a better understanding of the infection dynamics of influenza viruses in their natural reservoir. This might ultimately lead to interventions that enhance our pandemic preparedness.

## INTRODUCTION

Waterfowl are considered the natural reservoir for avian influenza viruses.^1, 2^ While it is known that a wide variety of influenza viruses circulate in wild birds, little is understood about the infection dynamics of influenza viruses within avian populations. There is evidence for seasonal patterns of influenza virus infections among waterfowl.^3, 4^ The mechanisms underlying these seasonal patterns are not known, but one possibility is that they are driven by the induction of humoral cross protective immunity after early infections.^5, 6^ Given the capacity for influenza viruses to spread via migratory avian hosts, as observed when H5N8 spread across North America in 2015,^7^ it is important to study the mechanics underlying these seasonal dynamics. This is specifically interesting in light of cross-reactivity/cross-protection within group 1 (H1, H2, H5, H6, H8, H9, H11, H12, H13, H16, H17 and H18) and group 2 (H3, H4, H7, H10, H14 and H15) hemagglutinnin (HA) expressing viruses by stalk-reactive monoclonal antibodies isolated from humans and mice.^8, 9^

The current techniques of influenza virus serology include assays such as the hemagglutination inhibition assay (HI), microneutralization assay (MN) and enzyme-linked immunosorbent assay (ELISA). While these techniques are useful and broadly accepted for serological analyses, they have significant limitations in the context of measuring the humoral response to the numerous subtypes and strains of influenza viruses. Assays that can measure the breadth of the antibody response to infection or vaccination without onerous labor requirements or need for large sample volumes are needed. Recently, researchers have started to develop protein microarrays for influenza virus serology.^10, 11, 12, 13, 14, 15, 16, 17, 18, 19, 20^ Protein microarrays are a high-throughput assay that can measure the magnitude and breadth of an antibody response. Like the ELISA (and unlike HI and MN assays), protein microarrays can also detect non-neutralizing antibodies, which are able to protect against influenza virus through effector functions such as antibody-dependent cell-mediated cytotoxicity and antibody-mediated cellular phagocytosis.^21, 22, 23, 24, 25^

**Figure 1:**
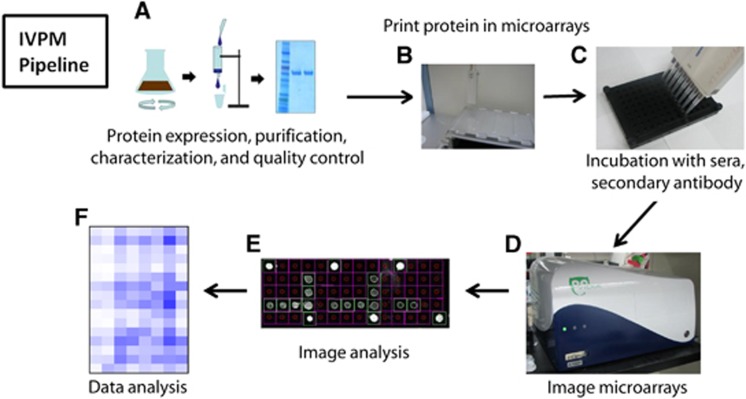
Influenza virus protein microarray pipeline. Recombinant HA is expressed in a baculovirus expression system, purified, characterized and undergoes quality control (**A**). HAs are arrayed onto an epoxysilane-coated glass slide (Schott) using a Versa 110 spotter (Aurora Biomed) (**B**). The HAs are covalently bound to the slide and blocked; the arrays are incubated with sera, followed by fluorescently labeled secondary antibodies in a 96-well microarray gasket (Arrayit) (**C**). The arrays are then imaged (**D**), spots are automatically detected and their fluorescence is measured (**E**). Data from microarray imaging are analyzed in GraphPad Prism 7.0 (**F**). HA, hemagglutinin.

To that end, we analyzed sera from mallard ducks experimentally inoculated with two low-pathogenic avian influenza viruses, H3N8 and H4N5. A wide range of influenza virus-subtypes infect wild birds, and in order to understand the breadth of the humoral response in that context, we used representative recombinant HAs from each known subtype in our influenza virus protein microarray (IVPM). The IVPM, while very similar in concept to a conventional ELISA (Figure 1), boasts superior throughput. In this study, one 96-well gasket loaded with microarrays yielded as much reactivity data as 24 ELISA plates. IVPMs also use six times less sera, and 37 times less recombinant HA compared to ELISAs to obtain equivalent reactivity data. Because this technology is relatively new, we validated it against ELISA. After validating the IVPM, we demonstrated its utility by analyzing a set of sera from wild mallards. This technology will allow researchers to efficiently interrogate large sample sets for reactivity to a wide variety of viral antigens and will be a powerful tool to assess the breadth of the humoral response to vaccination and infection in multiple species.

## MATERIALS AND METHODS

### Recombinant protein

Recombinant HAs from each influenza A subtype (Table 1) were produced in a baculovirus expression system. Recombinant baculoviruses expressing soluble HAs with trimerization domains and a hexahistidine tag were propagated in an Sf9 insect cell line (ATCC# CRL-1711), then used to infect BTI-*TN*-5B1-4 cells, which are better suited to secretion of the recombinant HA. Recombinant HA was purified from the supernatant on Ni-nitrilotriacetic acid resin columns as described previously.^26, 27^

### Antibodies

Anti-duck IgY antibody was labeled using a Cy3 Fast Conjugation Kit (Abcam), as per the manufacturer’s protocol. Secondary antibodies used included horseradish peroxidase (HRP) linked anti-bird IgY (Novus Biologicals, Littleton, CO, USA, catalog number NB7228, lot P21; Novus), HRP-linked anti-duck IgY (KPL, Gaithersburg, MD, USA, 04-25-06, lot 150077; KPL), HRP-linked anti-duck IgY (Antibodies Online, catalog number ABIN457698, lot 6478; AbO) and HRP-linked anti-mouse IgG (Rockland, 610-603-002, lot 33651). KB2, a monoclonal antibody (mAb) that is conformation-sensitive and binds the stalk domain of group 1 influenza A HAs^28^ was also used.

### ELISA

Ninety-six-well Immulon 4 HBX plates (Thermo Scientific, Waltham, MA, USA) were coated with recombinant HA proteins at 2 μg/mL (50 μL/well) in coating solution (KPL) overnight. The coating solution was removed, and the wells were blocked with 220 μL/well 3% nonfat milk in phosphate-buffered saline (PBS) containing 0.1% Tween 20 (PBST) for 1–3 h at room temperature. After removing the blocking solution, mallard sera were added at a starting concentration of 1:200 in 1% milk PBST, serially diluted 1:2 10 times in 1% milk PBST and incubated for 1 h at room temperature. After the sera were removed, and the plates were washed three times with 300 μL/well PBST, 100 μL secondary antibody solution (HRP labeled anti-IgY antibody from AbO, Novus or KPL, diluted 1:3000 in 1% milk PBST) was added to each well. After 1 h incubation period, the secondary antibody solution was removed and plates were washed four times with 300 μL/well PBST, and 100 μL/well SigmaFast OPD (*o*-phenylenediamine dihydrochloride (Sigma, St Louis, MO, USA)) was added. After 10 min, 50 μL/well 3 M HCl was added, and the optical density of each well was measured at 490 nm with a Synergy H1 hybrid multimode microplate reader (BioTek, Winooski, VT, USA). Area under the curve (AUC) was measured as total peak area above a baseline of three standard deviations above the mean optical density of background wells. Background wells were coated with recombinant HA, blocked and incubated with secondary antibody, but not incubated with sera.

ELISAs with mAbs were performed as above, but using an anti-mouse IgG antibody (Rockland). The starting concentration of the mAb KB2 was 30 μg/mL followed by twenty four 1:2 serial dilution steps.

### IVPM

Recombinant HA was spotted in arrays of 20 spots onto Nexterion E epoxysilane-coated glass slides (Schott, Mainz, Germany). Each array was comprised of six unique HAs, spotted in triplicate at a concentration of 100 μg/mL in PBS and at a volume of 30 nL per spot. Twenty-four arrays were spotted on each slide. After spotting, each slide was incubated for 90 min at >95% relative humidity in a sealed chamber at room temperature, allowing the HA to bind covalently to the slides. After removing the slides and allowing them to dry, slides were blocked with 3% milk in PBST for 1 h. Slides were then washed in PBST by immersion, and inserted into 96-well microarray gaskets (Arrayit, Sunnyvale, CA, USA), isolating each array. Mallard sera were added at a starting concentration of 1:50 in 1% milk PBST at a volume of 100 μL/array, and diluted two times at 1:10 into separate arrays and incubated for 1 h. After the sera were removed, arrays were washed three times with 220 μL/array PBST and then 50 μL secondary antibody solution (anti-duck IgY antibody (Antibodies Online) labeled with Cy3 fluorescent dye) was added to each array and incubated for 1 h. The secondary antibody solution was removed and slides were removed from 96-well microarray gaskets and washed in PBST by immersion. Slides were allowed to dry at room temperature, and analyzed for mean fluorescence using a Vidia microarray scanner (Indevr, Boulder, CO, USA), using an exposure time of 1300 ms. AUC was measured as total peak area above the mean fluorescence of spots of the same HA incubated with naive mallard sera.

### Experimental infection of mallards

Mallard (*Anas platyrhynchos*) sera used in the study were available from a previously published study.^5^ All work was approved by the University of Georgia Animal Care and Use Committee under AUP number A2013 05–021. Mallards were raised in captivity at the Animal Resources College of Veterinary medicine at the University of Georgia, and confirmed to be naive to influenza virus by nucleoprotein ELISA. Mallards were initially inoculated at an age of 4 weeks. Mallards receiving a second inoculum were inoculated 5 weeks later. The strains used were A/mallard/MN/SG-00169/07 (H3N8) and A/mallard/MN/AI11-4213/11 (H4N5). Blood samples were collected 2 weeks after single infections with H3N8 and H4N5. For the H3N8xH3N8 and H3N8xH4N5 groups, sera were collected 2 weeks after the final virus challenge. Each group of infected animals was made up of five or more mallards, with a male:female ratio of ~1:1. One mallard in the group inoculated with H3N8 and H4N5 was excluded because of atypically high reactivity.

### Statistical analyses

AUC was calculated in GraphPad Prism 7.0 (for Apple). When calculating AUC for ELISA results, AUC was measured above a baseline (defined as the mean optical density (OD) plus three times the standard deviation of background wells). For microarray results, AUC was measured as total peak area, using the mean fluorescence of naive sera as the baseline above which area was measured (baselines were determined for each recombinant HA). Fold induction was determined by dividing AUCs by the residual reactivity of naive sera exceeding the mean naive baseline level. Pearson correlation analyses, one-way ANOVA, and unpaired *t*-tests were performed in GraphPad Prism 7.0 *P*-values less than 0.05 were considered significant.

### Samples from wild mallards

All mallards were sampled at Agassiz National Wildlife Refuge located in Northwestern Minnesota during September 2012 and 2013. Seven samples were taken from hatch year (HY) animals and one sample was from an older after hatch year (AHY) animal. Virus isolation data for mallards sampled at the same time at this site were available for both years. Serum collection was approved by the University of Georgia Animal Care and Use Committee under AUP numbers A2010 06-101 and A2013 05-021.

## RESULTS

### The IVPM pipeline

Establishing the IVPM was a major goal of this study. The recombinant HAs used in this study were produced in a baculovirus expression system (Figure 1A), and spotted in arrays onto Nexterion E epoxysilane-coated glass slides (Schott) (Figure 1B). After binding the HAs to the slide and blocking, mallard sera followed by secondary antibody were incubated with the arrays in a 96-well microarray gasket (Arrayit) (Figure 1C). Arrays were imaged in a Vidia microarray scanner (Indevr) (Figure 1D), and data from the arrays were analyzed in GraphPad Prism 7.0 (Figures 1E and 1F).

### Validating the IVPM with monoclonal antibodies

**Figure 2:**
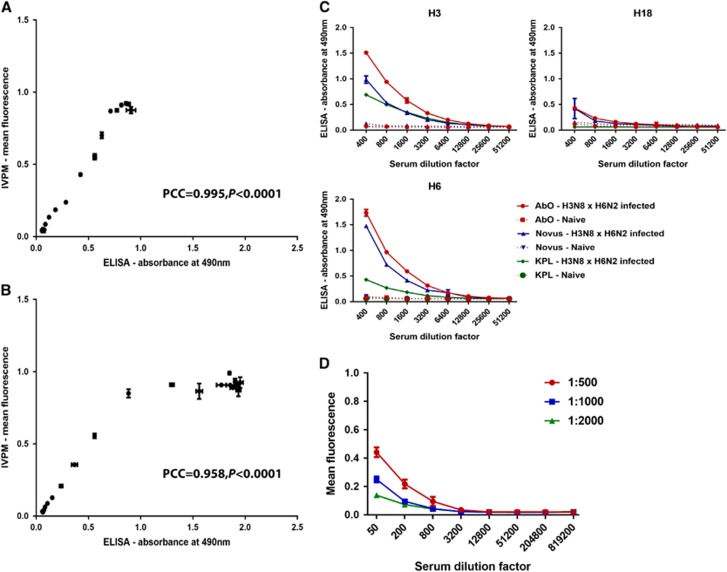
Establishing ELISA and IVPM for mallards. ELISA-IVPM correlation for serial 1:2 dilutions of mAb KB2, reacting to a conformational epitope on NC99 (**A**) and PR8 (**B**) H1 HA. The PCC and its *P*-value are indicated in both panels. (**C**) Testing of commercial secondary antibodies. Antibodies purchased from Antibodies Online (AbO), KPL (KPL) and Novus Biologicals (Novus) are shown, reacting against serum from a mallard infected with H3N8 and H6N2, binding H3, H6 and H18 HAs in ELISA. (**D**) Reactivity of different concentrations of pooled sera from mallards infected with H4N5 probed with different concentrations of the AbO secondary antibody in IVPM. The arrayed protein is recombinant H4. enzyme-linked immunosorbent assay, ELISA; influenza virus protein microarray, IVPM; Pearson correlation coefficients, PCC.

As a basic validation of the IVPM, we performed ELISA and IVPM assays using an identical dilution series of the murine mAb KB2 (Figures 2A and 2B), and measured binding to recombinant H1 HA from A/PR/8/34 (PR8) and A/New Caledonia/20/99 (NC99). KB2 is a conformation-sensitive mAb that binds the stalk region of the HA protein.^28^ The reactivity of KB2 to NC99 and PR8 HA by ELISA and IVPM correlated strongly, with Pearson correlation coefficients (PCC) of 0.995 (*P*<0.0001) and 0.958 (*P*<0.0001), respectively (Figures 2A and 2B). In the IVPM for PR8, concentrations of 0.029 μg/mL or above of KB2 produced mean fluorescence signals at the maximum level detectable by the microarray scanner, while OD in the ELISA continued to rise until the concentration of KB2 reached 0.234 μg/mL. For points in the dilution series in which signal was increasing in both assays performed with PR8, correlation was even stronger, with a PCC of 0.999 (*P*<0.0001).

### Validation of a commercial anti-duck antibody

Three commercially available HRP-linked antibodies that recognize duck IgY from Novus Biologicals (Novus), KPL (KPL) and Antibodies Online (AbO) were assessed for their ability to detect specific reactivity of duck serum to influenza virus HAs. Antibodies from H3N8- and H6N2-positive duck serum bound to recombinant H3, H6 and H18 HA were measured in ELISA, using the three secondary antibodies. The secondary antibodies from Antibodies Online and KPL were specified as duck-specific, while the antibody from Novus Biologicals was specific for sparrow, dove and chicken IgY in addition to duck IgY. The secondary antibody purchased from Antibodies Online yielded the greatest signal to background ratio of the three antibodies (Figure 2C), and was used in subsequent IVPM assays in a fluorophore-labeled version. The antibody purchased from KPL had the lowest reactivity to duck IgY by ELISA, while the Novus Biological antibody had marginally lower reactivity compared to the antibody from Antibodies Online (Figure 2C). Given that this antibody also binds to immunoglobulins of other avian species, it may be of interest in future studies incorporating multiple avian species.

In order to determine a suitable starting concentration of sera and concentration of secondary antibody to use in the microarray, we tested serially diluted pooled mallard sera from animals infected with H4N5 on an IVPM including recombinant H4. The IVPM was subsequently incubated with three different dilutions of secondary antibody (Figure 2D). We selected the highest concentrations of secondary antibody (1:500) and starting serum dilution factor (1:50), which produced a mean fluorescence in the middle of our detecting range.

### Analysis of sera from mallards experimentally infected with influenza viruses

**Figure 3:**
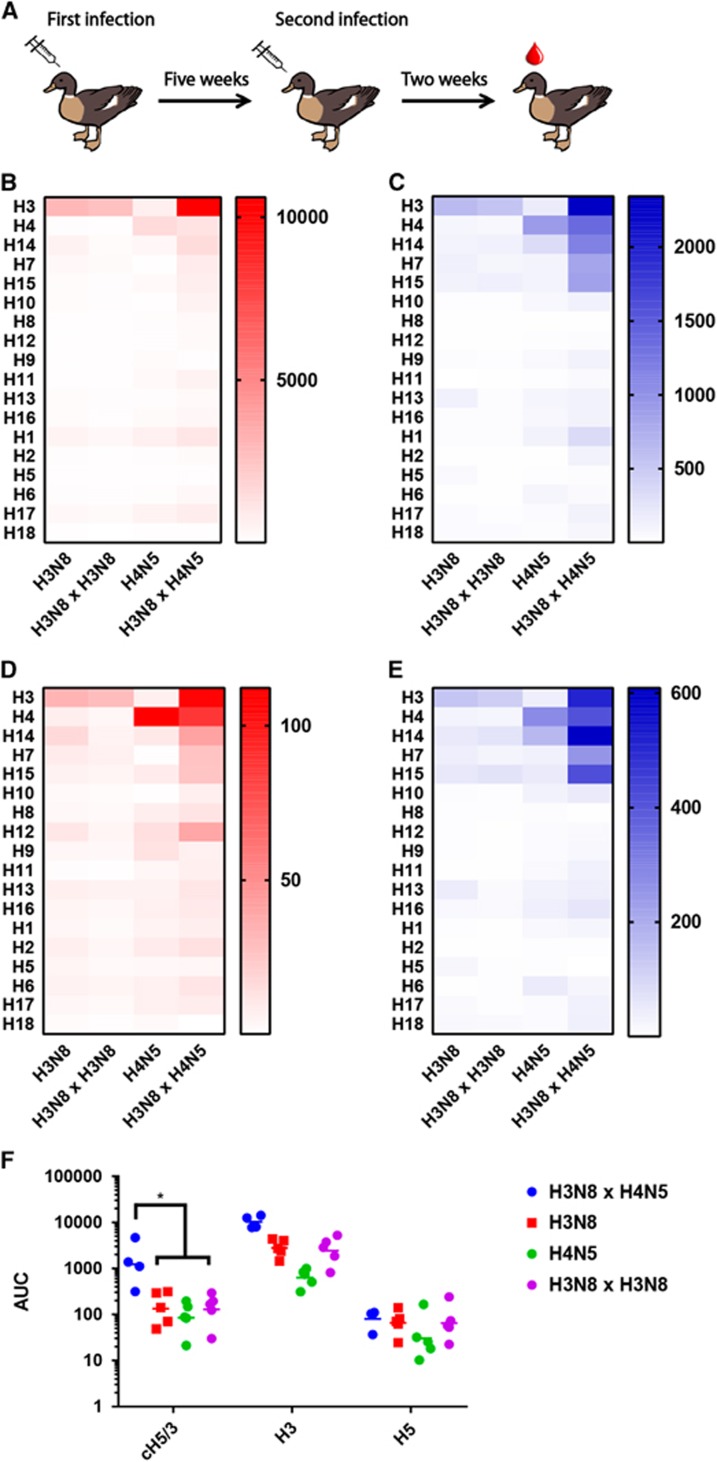
Reactivity of sera from experimentally infected mallards against recombinant HA in ELISA and IVPM. Infection and sample collection scheme for experimentally inoculated mallards (**A**). Absolute AUC values (**B**) and fold induction (**C**) over the AUC of naive sera against recombinant HA in ELISA are shown, calculated for each HA. (**D**) AUC and (**E**) fold induction data collected via the IVPM. (**F**) Reactivity of sera to chimeric H5/3 (cH5/3), H3 and H5 in ELISA. area under the curve, AUC; enzyme-linked immunosorbent assay, ELISA; hemagglutinin, HA; influenza virus protein microarray, IVPM.

Sera collected from the experimental groups of mallard ducks infected with H3N8, H4N5, infected twice with H3N8 in an interval of 5 weeks or infected with H3N8 and 5 weeks later with H4N5 (Figure 3A) displayed similar trends in reactivity when assayed by ELISA and IVPM (Figures 3B–3E). Ducks inoculated once, or twice with H3N8, produced an antibody response that was specific to H3, with very limited cross reactivity to the other recombinant HAs on the panel (Figures 3B and 3C). Ducks inoculated once with an H4N5 virus showed a similar response, specific for H4. Ducks infected with H3N8 and later infected with H4N5 produced a stronger response against recombinant H3 than ducks that were only inoculated with H3N8, as well as higher reactivity to non-H3 or H4 group 2 HAs (*P*=0.0004) (unpaired *t*-test of IVPM data). Very similar results are observed in both IVPM and ELISA when fold-induction over naive sera (Figures 3D and 3E) is analyzed.

To investigate possible mechanisms for this cross-reactivity, a chimeric HA incorporating the head domain of the H5 A/Vietnam/1203/04 strain (a group 1 HA) and the stalk of A/Perth/16/09 H3 (cH5/3)^29^ was used in ELISA to specifically measure the anti-stalk humoral response to infection with H3N8 and H4N5 (Figure 3F). Sera from ducks infected with H3N8 and H4N5 showed higher reactivity to chimeric H5/3 than any other group (one-way ANOVA, *P*<0.05), indicating a relatively high induction of anti-stalk antibodies in that experimental group. A similar phenomenon has been described previously in mammals (including humans and mice) sequentially infected or vaccinated with divergent influenza A viruses.^30, 31, 32, 33^

All of the experimentally infected animals tested showed seroconversion by ELISA and IVPM. The majority of these samples (24 out of 29) were also tested by nucleoprotein (NP) competition ELISA as well as microneutralization assays. All but two of the tested animals seroconverted as measured by NP ELISA. One of the two negative NP ELISA samples was positive in MN assay, the second one was also negative by microneutralization assay. While this does not allow strong conclusions, it seems that both ELISA and IVPM show more sensitivity than NP ELISA and the microneutralization assay.

### Correlation analysis

**Figure 4:**
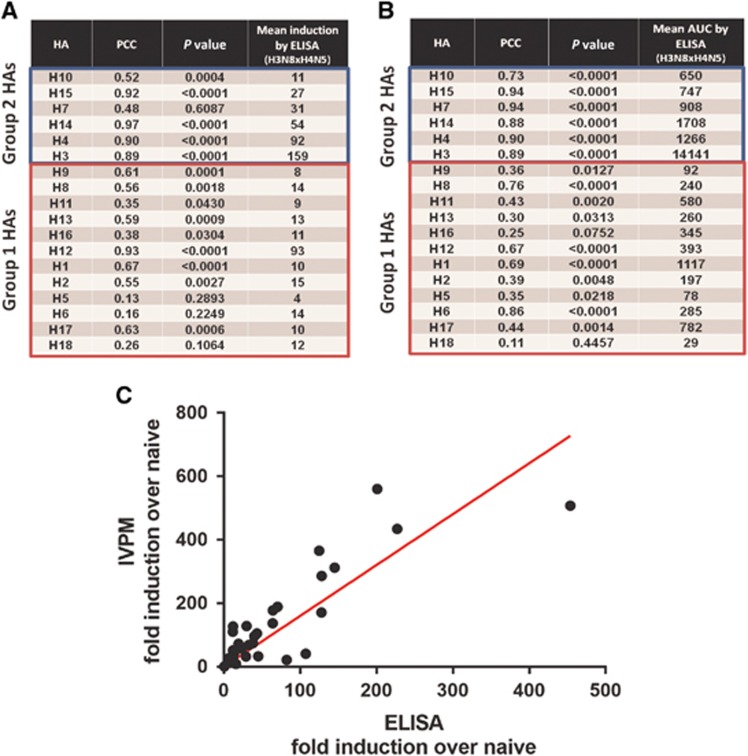
Correlation analysis of reactivity data measured by IVPM and ELISA. Correlation between IVPM and ELISA fold induction over naive sera (**A**) and IVPM and ELISA absolute AUC values (**B**) are shown, by HA. Correlation of ELISA and IVPM fold induction data for recombinant H3 shown as an example of the correlation analysis (**C**). area under the curve, AUC; enzyme-linked immunosorbent assay, ELISA; hemagglutinin, HA; influenza virus protein microarray, IVPM.

To assess the comparability of the ELISA and IVPM, Pearson correlation analyses were performed for each recombinant HA (Figure 4). Correlation between ELISA and IVPM was strong within group 2 HAs, where overall reactivity was higher, relative to group 1 HAs. For all recombinant HAs in group 2, PCCs for ELISA and IVPM absolute AUC values were between 0.73 and 0.94 (*P*<0.0001) (Figure 4B). Correlation between fold-induction values derived from ELISA and IVPM data was promising, but not as strong throughout—for H3, H4, H14 and H15, PCCs were 0.89, 0.90, 0.97 and 0.92 (*P*<0.0001), respectively (Figure 4A). An example of the analysis for H3 is shown in Figure 4C. The PCC for H10 results was 0.52, but also highly significant (*P*<0.0004). Correlation between ELISA and IVPM was less uniform among group 1 HAs, where reactivity was low. For some HAs, ELISA and IVPM results correlated well (that is, H1, PCC 0.67 (*P*<0.0001) for absolute AUC values and fold induction), but for other HAs in group 1, correlation was low (Figures 4A and, 4B). This is not unexpected since low reactivity might lead to a higher error. However, the correlation was highly significant even for most group 1 HAs.

### Analyzing wild mallard samples

**Figure 5:**
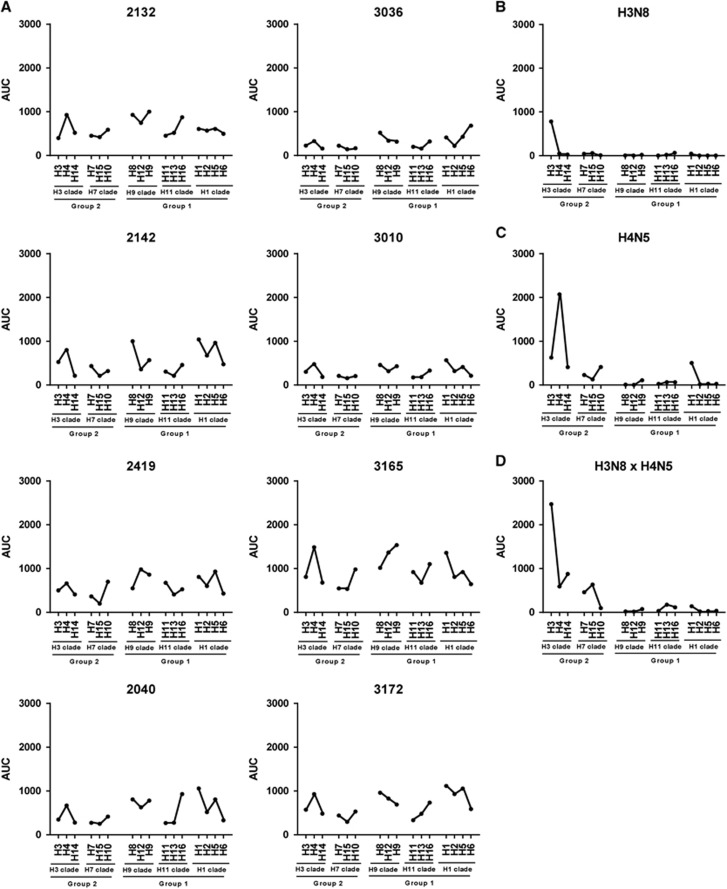
Reactivity data for serum samples from wild mallard ducks. (**A**) Reactivity profiles of eight serum samples from wild mallard ducks. HA subtypes, clades and groups are indicated on the *x*-axes of the panels. Sera from individual captive mallards experimentally infected with H3N8 (**B**), H4N5 (**C**) or H3N8 followed by H4N5 (**D**) are shown as comparison. hemagglutinin, HA.

To test if the IVPM is able to detect sero-reactivity in samples from wild mallards, we tested eight samples that were collected during surveillance at the Agassiz National Wildlife Refuge (Minnesota, USA) in 2012 and 2013. Samples included seven samples from HY animals and one sample from an older AHY animal. The eight wild mallard samples analyzed displayed a variety of antibody repertoires, suggesting a diversity of infection histories (Figure 5A). Samples 3036 and 3010 had low reactivity overall, with moderate or low reactivity toward 2–4 HAs (H1, H4, H6, H8 and H9) included in this panel. The six other samples analyzed had moderate or high reactivity to five or more HAs with distinct peaks. Peak reactivity toward several HAs was observed across all samples, with H1, H4, H5 and H8 being the most prevalent ones. Sample 3165, which was from the only AHY animal, included the four highest reactivity values observed, against H9, H4, H12 and H1 (in that order). This suggests that wild mallards are likely exposed to a variety of influenza subtypes in their habitat. Obviously, this complicates the analysis since—as shown for the experimentally infected birds—sequential exposure to different subtypes broadens the immune response. This might explain to some extent the higher baseline reactivity in wild mallard samples as compared to samples from captive mallards in controlled infection experiments (Figures 5B–5D). In addition, it seems that experimentally infected animals developed higher titers against the strain they were infected with (with a lower background reactivity) than wild mallards, but this is likely an artifact of timing since samples were taken from experimentally infected ducks shortly (2 weeks) after infection. The interval between infection and sampling in wild mallards is unknown and could be relatively long (months to years).

## DISCUSSION

Avian influenza viruses are a potential pandemic threat,^9^ and have demonstrated an ability to spread geographically with migrating birds.^34^ In addition to being a potentially grave threat to human health, highly pathogenic avian influenza viruses regularly infect poultry flocks, necessitating expensive culling.^35^ Understanding the dynamics of influenza virus infections within populations of wild birds could be key to mitigating these threats and serological assays could help to collect crucial information for this purpose.

Mallards inoculated with only one influenza A virus (H3N8 or H4N5) produced a specific humoral response to the HA of the virus they were inoculated with. Among mallards inoculated twice with H3N8, reactivity to H3 was not significantly boosted after the second inoculation with H3N8, likely because the mallards developed sterilizing immunity to the virus after the first exposure. The animals in this study were infected with A/mallard/MN/SG-00169/07 (H3N8), while the H3 protein available to use for detection in this study was derived from A/harbor seal/Massachusetts/1/11 (H3N8) (Table 1). These HAs share 97.1% of their amino-acid sequence so it is expected that most strain-specific antibodies would have been detected. In contrast to sequential inoculation with H3N8, inoculation of H3N8-infected animals with H4N5 led to a dramatic change in antibody repertoire, including a strongly boosted response to H3 and a broadening of the reactivity to other group 2 HAs, with a proportion of the response being driven by antibodies to the conserved stalk of HA. Interestingly, it has been shown that the degree of protection of mallard ducks after a secondary infection with a different virus subtype correlates to the similarity between HAs^5^ and may be the result of reactivity of anti-stalk antibodies. The induction of broadly reactive anti-stalk antibodies has been reported in mice and humans that were sequentially exposed to different HA subtypes of the same group ^30, 32, 33, 36, 37, 38, 39^ (for example, H1 and H5), but this phenomenon has not been reported so far in avian species.

The antibody repertoire of the wild mallard sera collected at Agassiz National Wildlife Refuge was highly reactive to many HA subtypes in six out of eight samples. Reactivity to H1, H4, H5, H8 and H9 was common to all samples, suggesting the circulation of viruses containing HAs belonging to these subtypes, and a potential resistance to future infections with those viruses among the sampled animals. It is interesting that even relatively young birds, which had been born earlier in the year the samples (HY) were taken, showed reactivity. The highest reactivity, however, was found in a sample from an older (AHY) animal. Virus isolation results from mallards at this site during those years documented the presence of viruses representing multiple influenza A virus HA subtypes. During 2012, 134 viruses were isolated representing H1, H2, H3, H4, H5, H10, H11 and H12 subtypes. In 2013, there were 93 viruses isolated (H1, H2, H3, H4, H11 and H12). Although H8 and H9 influenza A viruses were not isolated in either year, this may represent a limitation of sampling. During both years, sampling was restricted to September and previous work at this location reported that influenza A viruses, often of different HA subtypes that are encounter during September, can be isolated as early as July.^40^ We also found that samples from wild mallards have higher baseline reactivity than samples from captive birds after experimental infection. This might be caused by the broadening of the immune response following sequential infection with different influenza viruses. In addition, reactivity to the matched HA was higher in experimental infection than in samples from wild mallards, likely because samples from captive birds were obtained shortly after infection before immune responses start to decay. The lower signal and higher background might make it more difficult to infer infection history in sera from wild avian species. However, it was possible to see clear peaks in the reactivity profiles that indicated infection with specific subtypes. Future experiments that estimate antibody waning and potentially the computational analysis of larger datasets generated from samples from experimentally infected and wild birds might help to develop a methodology that allows to infer a more exact infection history.

The IVPM is a powerful serological assay for addressing the question of infection history and circulation dynamics of avian influenza viruses in the avian reservoir. The results obtained in this study by IVPM correlate well with those obtained by ELISA, providing a solid basis for this new methodology to be considered a useful serological assay. The IVPM offers a significant increase in throughput and allows more data to be obtained from smaller volumes of sera, compared to the ELISA. In this study, six times less sera and thirty-seven times less recombinant HA was needed to obtain reactivity data by IVPM compared to ELISA, and we are continuing to improve these advantages in reagent efficiency. Current IVPMs yield thirty-two times more reactivity data from each 96-well gasket loaded with microarrays when compared to an ELISA microwell plate, and now use eight times less sera than ELISAs for equivalent experiments. AUC analysis on fluorescence data from microarrays incubated with serial dilutions of sera was key to obtaining accurate measures of reactivity, especially in cases of signal saturation at higher serum concentrations. Serial dilution steps ensured that each sample could be analyzed within the dynamic range of the microarray scanner. While this study is focused on mallard ducks, the IVPM can be used to interrogate sera from a wide variety of influenza virus host species, animal models and humans, and is limited only by the availability of appropriate secondary antibody. Adapting this assay to assess reactivity to antigens from other pathogens of interest alongside or instead of influenza virus proteins is also possible, much in the same way that the ELISA has broad usefulness.

## Figures and Tables

**Table 1:** Recombinant HAs

**HA subtype**	**Virus from which the recombinant HA was derived**
H1	A/South Carolina/1/18 (H1N1)
H1	A/Puerto Rico/8/34 (PR8, H1N1)
H1	A/New Caledonia/20/99 (NC99, H1N1)
H2	A/mallard/Netherlands/5/99 (H2N9)
H3	A/harbor seal/Massachusetts/1/11 (H3N8)
H4	A/duck/Czech/56 (H4N6)
H5	A/Vietnam/1203/04 (H5N1)
H6	A/mallard/Sweden/81/02 (H6N1)
H7	A/chicken/BC/CN-6/04 (H7N3)
H8	A/mallard/Sweden/24/02 (H8N4)
H9	A/chicken/Hong Kong/G9/97 (H9N2)
H10	A/mallard/Interior Alaska/10BM01929/10 (H10N7)
H11	A/shoveler/Netherlands/18/99 (H11N7)
H12	A/mallard/Interior Alaska/7MP0167/07 (H12N5)
H13	A/black headed gull/Sweden/1/99 (H13N6)
H14	A/mallard/Gurjev/263/82 (H14N5)
H15	A/shearwater/West Australia/2576/79 (H15N9)
H16	A/black headed gull/Sweden/5/99 (H16N3)
H17	A/yellow shouldered bat/Guatemala/06/10 (H17N10)
H18	A/bat/Peru/33/10 (H18N11)
